# Correlation between thymidylate synthase gene polymorphisms and efficacy of pemetrexed in advanced non-small cell lung cancer

**DOI:** 10.3892/etm.2012.730

**Published:** 2012-09-28

**Authors:** QIONG HU, XUEFEI LI, CHUNXIA SU, XIAOXIA CHEN, GUANGHUI GAO, JIE ZHANG, YINMIN ZHAO, JIAYU LI, CAICUN ZHOU

**Affiliations:** 1 Department of Medical Oncology, Shanghai Pulmonary Hospital, Tongji University School of Medicine;; 2 Tongji University Medical School Cancer Institute, Shanghai 200433, P.R. China

**Keywords:** non-small cell lung cancer, pemetrexed, thymidylate synthase, polymorphism

## Abstract

One of the target genes of pemetrexed (PEM), thymidylate synthase (TS), has been shown to have a close association with its efficacy. TS gene polymorphisms have been shown to be associated with the efficacy of antifolate treatment in enteron tumors. The purpose of this study was to investigate the clinical significance of TS gene polymorphisms in patients with advanced NSCLC receiving PEM-based treatment. The variable nucleoid tandem repeat in the 5′-UTR region was amplified and detected using fluorescently labeled multiplex short tandem repeat polymerase chain reaction. The polymorphism in the 3′-UTR region of the TS gene was detected using the Taqman probe. Efficacy of PEM was assessed according to the Response Evaluation Criteria in Solid Tumors, version 1.1. None of the genotypes were associated with gender, smoking status and age. Disease control rate (DCR), objective response rate (ORR) and progression-free survival (PFS) were similar between patients harboring 2R and 3R alleles (PFS, p=0.518; DCR, p=0.631; ORR, p=0.541), as well as those with a 6-bp insertion and 6-bp deletion (PFS, p=0.776; DCR, p=0.626; ORR, p=0.330). To study the combined effect of TS polymorphisms, the study population was divided into three groups: 2R&6 del, 2R&6 ins and 3R&6 del. No significant differences were observed among the different groups according to DCR (p=0.517), ORR (p=0.611) and PFS (p=0.938). In conclusion, polymorphisms of the TS gene do not appear to be a prognostic marker for advanced NSCLC patients receiving PEM-based treatment.

## Introduction

Thymidylate synthase (TS), as the *de novo* source of thymidylate synthesis, is an essential enzyme involved in DNA replication and cell growth (1). It catalyzes the conversion of deoxyuridylate (dUMP) to deoxythymidylate (dTMP), which is critical for DNA synthesis and repair. The substrate for TS is a central metabolite in folate metabolism. Due to its important role in the folate biosynthesis pathway, it has become one of the major targets of antitumor agents, such as 5-fluorouracil (5-Fu) and pemetrexed (PEM).

PEM is a multitarget antifolate agent that has produced excellent clinical outcomes in first-line, second-line and maintenance treatment in advanced non-small cell lung cancer (NSCLC) (2–4). Notably, PEM-based treatment provided more favorable clinical outcomes in patients with adenocarcinoma (ADC) compared with those with squamous cell carcinoma (SCC). A post hoc analysis of three randomized global trials confirmed the superiority of PEM in non-squamous non-small cell lung cancer (NSNSCLC) (5). A plausible explanation for this superiority may involve the expression of TS in different histological types. A Japanese study (6) collected 2621 NSCLC patients and examined TS expression in postoperative tissue samples obtained from this population. TS expression was categorized according to TS/β-actin values. In univariate analysis, TS gene expression in formalin-fixed and paraffin-embedded (FFPE) tumor samples was higher for SCC (mean TS/β-actin 4.3), compared with ADC (mean TS/β-actin 2.3) (p<0.01). Another study also confirmed that the mean scoring of TS was significantly higher in the non-ADC than in the ADC subgroup (2.79±0.61 vs. 1.98±0.88, p<0.0001) (7). Notably, the above analysis revealed that patients with positive expression of TS had a lower 5-year progression-free survival (PFS) rate than those presenting negative expression of TS (48.6 vs. 79.1%, p<0.0001). The 5-year overall survival (OS) rate was also significantly lower in the positive group (67.5 vs. 86.1%, p=0.0002). Among patients with ADC, the 5-year PFS rate was 30.5% in the TS expression positive group and 83.1% in the negative group (p<0.0001). The 5-year OS rates were 61.1 and 90.1%, respectively (p<0.0001). Sun *et al* (8) also demonstrated an association between a higher response rate for PEM-based chemotherapy and TS-negativity (33.7 vs. 14.1%, p=0.002). PFS for PEM-based treatment was significantly longer in the ADC population (2.9 vs. 1.4 months, p=0.001) and TS-negative subgroup (4.1 vs. 2.0 months, p=0.001). Multivariate analysis revealed that TS-negativity was associated with longer PFS [hazard ratio (HR), 0.70; 95% confidence interval (CI), 0.51–0.97].

The functional polymorphisms in the TS gene have been suggested to be a regulator of downstream protein expression and mRNA level (9,10). The most closely studied polymorphisms have focused on the variable number of tandem repeats (VNTR) of a 28-bp sequence (2R/3R) in the 5′-untranslated region (UTR), a single nucleotide polymorphism (SNP) in the second repeat of 3R allele (G>C) and a 6-bp deletion or insertion in 3′-UTR of the TS gene. Preclinical study revealed that the triple repeat occurring in 5′-UTR plus the 6-bp insertion conferred a higher transcriptional efficiency and greater mRNA stability than the double repeat plus 6-bp deletion *in vitro* (10,11). The association between these polymorphisms and efficacy of 5-Fu-based chemotherapy has been demonstrated in certain solid tumors, such as gastric, colorectal and breast cancer (12–14). A previous study suggested that VNTR and SNP in the 5′-UTR of the TS gene in combination with a C667T polymorphism of methylenetetrahydrofolate reductase (MTHFR) were associated with prognosis of NSCLC (15). However, there was no difference in prognosis between different genotypes when TS and MTHFR groups were considered separately.

Based on the data mentioned above, the present study was conducted to further investigate the association between polymorphisms of the TS gene and efficacy of PEM-based treatment in advanced NSCLC.

## Materials and methods

### Patients

This study was approved by the ethics and research committee of our hospital. Data were obtained from 90 patients presenting with advanced NSCLC receiving PEM-based treatment between March 2009 and September 2011 at the Department of Medical Oncology in Shanghai Pulmonary Hospital. Diagnoses were confirmed by cytological or histological examinations. The extent of disease was determined according to the 7th edition of the American Joint Committee on Cancer/International Union Against Cancer (AJCC/UICC) classification (16,17). Non-smokers were defined as having smoked fewer than 100 cigarettes in their lifetime and without use of other tobacco products.

### DNA extraction and genotyping

Peripheral blood samples were drawn into ethylene diamine tetra-acetic acid (EDTA)-containing tubes and preserved at −20°C in a refrigerator for final test. Genomic DNA was extracted from 100 μl of blood using the Takara genomic DNA extraction kit (Takara Biotechnology, China). Polymorphisms were detected by Taqman probes designed and synthesized by Invitrogen. The sequences of probes and primers are listed in Table I. PCR analysis was performed as previously reported (9). PCR products were confirmed by capillary electrophoresis) and then detected using a 3730XL DNA detection device (Applied Biosystems, USA). Polymerase chain reaction (PCR) for TS 3′-UTR genotyping was carried out in a volume of 20 μl solution containing 1.0 μl genomic DNA, 9.7 μl ultrapure water, 2 μl 10X PCR buffer, 1.2 μl 50 mmol/l MgCl_2_, 0.5 μl 10 mmol/l (10 mM) dNTPs, 4 μl 5X GC buffer, 0.5 μl of each primer (10 μM), 0.2 μl of probe-FAM (10 μM), 0.2 μl of probe-VIC (10 μM) and 0.2 μl 5 U/μl Taq DNA polymerase (Invitrogen). Cycling parameters were as follows: pre-PCR at 50°C for 1 min; held at 95°C for 10 min; 40 cycles of 95°C for 15 sec, 60°C for 1 min; and post-PCR at 60°C for 1 min. PCR was performed using QPCR 7500 fast instrument supplied by Applied Biosystems (USA). PCR products were analyzed using a real-time fluorescent PCR allele discrimination system for genotyping.

### Treatment

Each patient received at least one cycle of PEM-based treatment. PEM was administered at 500 mg/m^2^/cycle every 3 weeks with platinum regimens as first-line treatment or a single-agent treatment in second-line or further. All patients received premedication with folic acid (0.4 mg/day) and vitamin B12 (1 mg intramuscularly every 9 weeks). Oral corticosteroids (fluorodexamethasone 4 mg, twice/day) and ranitidine (0.15 g, twice/day) were administered on the day before, the day of and the day after chemotherapy. The number of cycles was documented.

### Evaluation of efficacy

The Response Evaluation Criteria in Solid Tumors (RECIST) version 1.1 was used to assess the efficacy independently (18). Complete response (CR) was defined as the disappearance of all target lesions as well as any pathological lymph nodes (whether target or non-target) reduced to <10 mm in short axis. Partial response (PR) was defined as a decrease of no less than 30% in the sum of diameters of target lesions compared with the baseline sum diameters. Progressive disease (PD) was defined as an increase of no less than 20% in the sum of diameters of target lesions compared with the smallest sum. In addition to the relative increase of 20%, the sum must also demonstrate an absolute increase of at least 5 mm. The appearance of one or more new lesions was also considered progression according to the new criteria. As for non-target lesions, the assessment was also performed complying with RECIST version 1.1.

### Statistical analyses

The effect of each TS polymorphism was assessed independently and in combination. 5′ polymorphisms were categorized as 2R if the genotype contained a functional 2R allele (2R/2R, 2R/3R) and 3R if the genotype contained a 3R allele (3R/3R, 3R/4R) (9). 3′ polymorphisms were categorized as ins6 if the genotype was ins6/ins6 and del6 if genotypes were ins6/del6 and del6/del6 (19). The combined effects of TS polymorphisms were analyzed by grouping the 5′ and 3′ genotypes, yielding three categories, 2R&del6, 2R&ins6 and 3R&del6. The statistical analyses were performed using SPSS version 16. The interval between dates of first administration of PEM and first recorded disease progression, unacceptable toxicity or last visit was defined as progression-free survival (PFS). The date of the last visit was December 30, 2011. Chi-square test and Pearson’s test were used to carry out univariate analyses. Survival curves were generated by the Kaplan-Meier method and statistical differenceswere evaluated using the log-rank test. The significance level of p-value was set at p<0.05.

## Results

### Patients and polymorphisms

**Figure 1:**
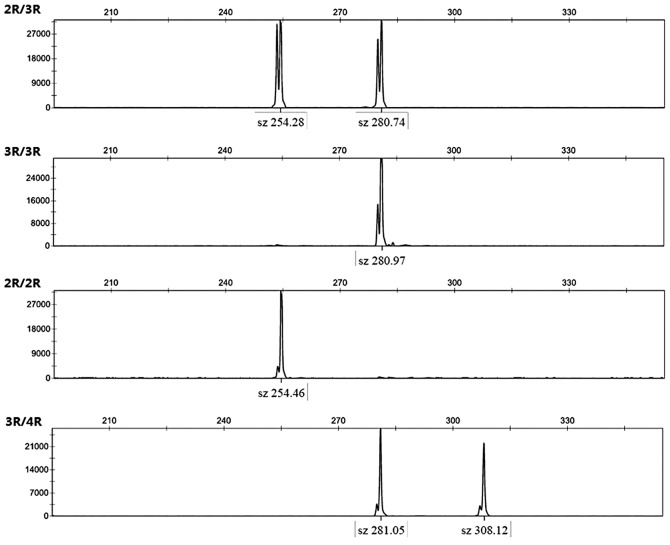
Four different genotypes were detected in the 5′-UTR of the TS gene.

**Figure 2:**
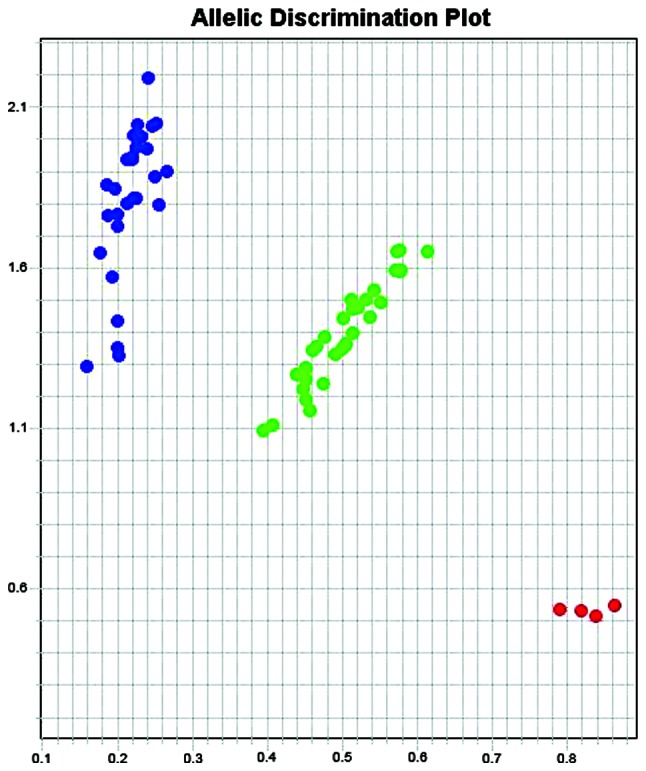
Different genotypes detected using the discrimination system. Allele 1 (red dot) represents the ins6/ins6 genotype. Allele 2 (blue dot) represents the del6/del6 genotype. Green dots represent the ins6/del6 genotype.

A total of 90 patients were studied and their baseline features are listed in Table II. The majority of the patients were under 70 years of age (79/87.8%). Each gender represented half of the study population. There was a dominant proportion (86/95.6%) of stage IV NSNSCLC. The number of non-smokers was greater than that of smokers, accounting for 62.2% of the entire group. The majority of patients (56/62.2%) received PEM in second-line or further. The median cycle of PEM administration was 4. At the final cut-off date (December 30, 2011), more than 80% of the patients had experienced progressive disease. Four types of VNTR in the 5′-UTR region of the TS gene were detected (Fig. 1). The discrimination system detected three polymorphisms located in the 3′-UTR region (Fig. 2). Among the study population, 2 patients exhibited 2R/2R (2.2%), 54 exhibited 3R/3R (60%), 33 exhibited 2R/3R (36.7%) and one exhibited 3R/4R (1.1%). Distribution of the 3′-UTR polymorphism was: 40 (44.4%) cases of del6/del6, 43 (47.8%) of ins6/del6 and 7 (7.8%) of ins6/ins6. No differences were observed in the distribution of various genotypes according to age, gender, smoking status, line of treatment and cycles (Table III).

### Association between polymorphisms and clinical outcomes

**Figure 3:**
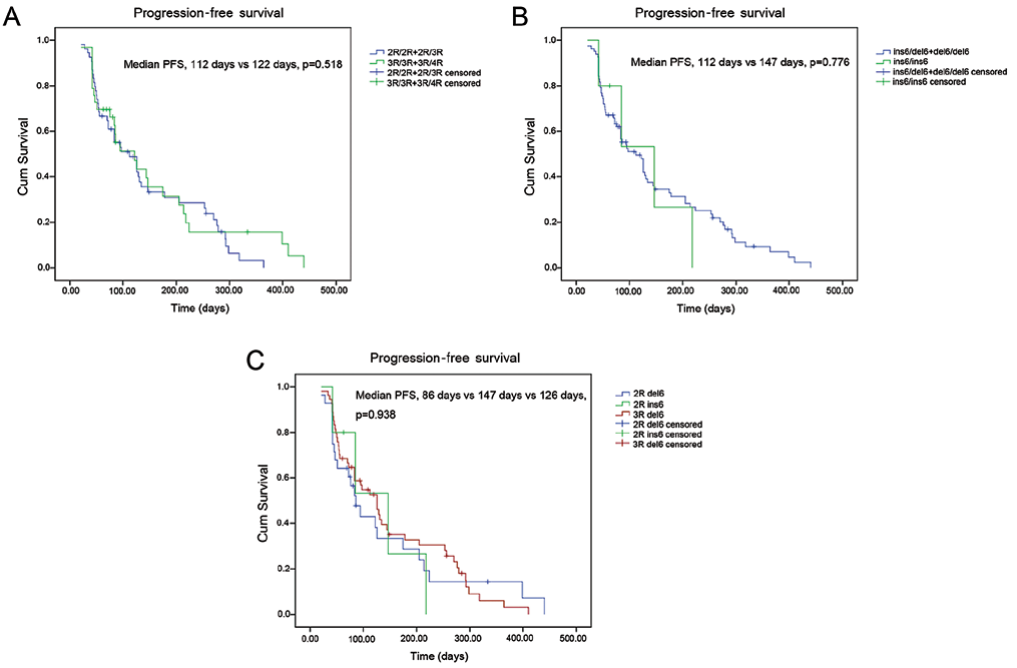
Progression-free (PFS) survival according to different genotypes. (A) Patients with 2R allele and 3R allele shared similar PFS (112 vs. 122 days, p=0.518). (B) No significant difference was observed between patients with ins6/del6 and del6/del6 polymorphisms and those with ins6/ins6 (112 vs. 147 days, p=0.776). (C) Combination of different genotypes did not yield significant differences (86 vs. 147 vs. 126 days, p=0.938).

All patients were available for evaluation of response rate and PFS. For the entire study population, the objective response rate (ORR) and the disease control rate (DCR) was 11.1 and 65.5%, respectively. Median PFS was 112 days. In the 5′-UTR group, patients harboring 2R alleles had similar clinical outcomes compared with those harboring 3R alleles (PFS, p=0.518; DCR, p=0.631; ORR, p=0.541), as shown in Table IV and Fig. 3A. In 3′-UTR group, no difference was observed between patients carrying 6- allele (del6/del6 and ins6/del6) and those carrying 6+ allele (ins6/ins6), as shown in Table IV and Fig. 3B. Median PFS were 112 and 147 days, respectively (p=0.776). Combination of genotypes did not yield significant differences among different groups. In 2R&del6, 2R&ins6 and 3R&del6 subset, DCR was 74.1, 57.1 and 62.5%, respectively (p=0.517). Accordingly, ORR was 11.1, 0 and 12.5% (p=0.611); median PFS was 86, 147 and 126 days (p=0.938) (Table IV and Fig. 3C).

## Discussion

In this study, we evaluated the correlation between polymorphisms in the TS gene and clinical outcomes of patients with advanced NSCLC following PEM-based chemotherapy. To our knowledge, this is the first study investigating the role of polymorphisms of the TS gene in PEM-treated advanced NSCLC patients. We demonstrated the lack of association between the polymorphisms and the efficacy of PEM-based treatment in advanced NSCLC. No significant differences were observed in ORR, DCR and PFS according to various genotypes. To our knowledge, this is the first study conducted in advanced NSCLC.

We noted an imbalance of stage and histological type in our study population. Due to the introduction of a new staging classification (16,17), some patients with malignant pleural effusion who were previously categorized as stage IIIb were re-categorized as stage IV. PEM is recommended for adenocarcinomas in first- and second-line treatment (20), and more than 90% of the study population presented with ADC. Due to the large disparity existing in these two subsets, we did not assess the association between polymorphisms and different stages or histological types.

Frequencies of TS polymorphisms reported by Takehara *et al* comprised 20 cases (6.8%) of 2R/2R, 75 (25.4%) of 2R/3R, 199 of 3R/3R and 1 of 3R/5R (67.8%) (15). Furthermore, TS VNTR did not show any significant association with clinicopathological factors in this Japanese study. The distribution of 3′-UTR polymorphism in a Chinese study was documented as follows: 7.6% ins6/ins6, 44.3% ins6/del6 and 48.1% del6/del6 (19). Based on the same ethnicity, our study also observed a similar distribution of the 5′-UTR polymorphism, with 2.2% of patients harboring 2R/2R, 60% harboring 3R/3R, 36.7% harboring 2R/3R and one with 3R/4R. The incidence of 3′-UTR polymorphisms was also consistent with those described in a previous publication (10). Our study has confirmed the lack of association between TS polymorphisms and clinical features in advanced NSCLC. However, when the imbalance of stage and histological type are taken into account, there may be deviations in the results, as the analyses were performed in a highly selected population. Therefore, our results may not serve as robust evidence.

Although TS VNTR and polymorphisms in the 3′-UTR region were demonstrated to be associated with efficacy of 5-Fu-based treatment, the majority of existing studies were performed in gastrointestinal cancers. Huang *et al* (21) collected 116 gastric cancer cases who received 5-Fu-based adjuvant chemotherapy. The study found a significant shorter overall survival (OS) in patients with TS ins6/ins6 genotype (20.7 months) compared with those with del6/del6 (29.8 months) (p=0.017) and ins6/del6 (41.0 months) (p=0.022) genotypes. Median relapse-free survival (RFS) was 11.5 months for the ins6/ins6 group, 20.8 for the ins6/del6 group and 36.9 for the del6/del6 subset. Another study on stomach cancer also found a significant correlation between polymorphisms in 3′-UTR of the TS gene and the sensitivity of gastric cancer to 5-Fu-based chemotherapy (19). The response rates of the del6/del6 and del6/ins6 groups were significantly higher than that of the ins6/ins6 group (p=0.045).

Notably, TS expression was demonstrated to be different in various histological types (8,9). The regulation of TS expression may be influenced by numerous uncertain factors (22). Due to the complicated communication between molecules occurring in the signaling pathways, it is assumed that the complex modulation of signaling pathways may also lead to a different expression level of TS in patients with tumors located in different organs and further result in different clinical outcomes. Gorlick *et al* reported a higher TS mRNA expression in pulmonary metastases (mean TS/β-actin ratio 19.7) when compared with hepatic lesions (mean TS/β-actin ratio 4.7) of colorectal cancer (23). Therefore, the TS expression in lung adenocarcinoma may differ from gastrointestinal cancers, which may also be a reasonable explanation for the converse results of our analysis and previous studies. However, we did not obtain data regarding TS expression. This was a limitation of our analysis in addition to the small scale of the study population.

In conclusion, our investigation demonstrated that the polymorphisms of the TS gene were unable to predict response to PEM-based treatment. However, this study was performed in a highly selected population. Therefore, further investigation should be conducted to yield a deeper insight into the overall value of these markers.

## Figures and Tables

**Table I:** Sequences of the primers and probes.

Primer/probes	Sequences (5′-3′)
Primers	
rs2853542	
Forward	GTGGCTCCTGCGTTTCCCCC
Reverse	GCGGAGGATGTGTTGGATCT
rs16430	
Forward	CGTGGACGAATGCAGAACACT
Reverse	TTCACAAGCTATTCCCTCAAATCT
Probes	
rs16430-del	FAM-ACAACTATAAAGTTCATAACCA-MGB
rs16430-CTTTAA	VIC-ATAACTTTAAAGTTCATAACC-MGB

**Table II:** Demographic and baseline characteristics of the study population.

Characteristics	No. of patients (%)
Overall	90 (100)
Age (years)	median 58; range, 34–78
≤70	79 (87.8)
>70	11 (12.2)
Performance status	
0/1	90 (100)
Gender	
Male	45 (50)
Female	45 (50)
Clinical stage	
IIIB	4 (4.4)
IV	86 (95.6)
Smoking status	
Non-smoker	56 (62.2)
Smoker	34 (37.8)
Line of treatment	
First-line	34 (37.8)
Second-line or further	56 (62.2)
Cycle	
≤4	64 (71.1)
>4	26 (28.9)
Histological type	
Adenocarcinoma	83 (92.2)
Non-adenocarcinoma	7 (7.8)
PD events	
PD	75 (83.3)
Non-PD	15 (16.7)

PD, progressive disease.

**Table III:** Association between polymorphisms and patient clinical features.

A, 5′-UTR			

Variable	2R/2R+2R/3R n (%)	3R/3R+3R/4R n (%)	P-value
Age (years)			
≤70	28 (35.4)	51 (64.6)	
>70	7 (63.6)	4 (36.4)	0.072
Gender			
Male	21 (46.7)	24 (53.3)	
Female	14 (31.1)	31 (68.9)	0.130
Smoking status			
Non-smoker	22 (39.3)	34 (60.7)	
Smoker	13 (38.2)	21 (61.8)	0.921
Line of treatment			
First-line	12 (35.3)	22 (64.7)	
Second-line or further	23 (41.1)	33 (58.9)	0.586
Cycle			
≤4	27 (42.2)	37 (57.8)	
>4	8 (30.8)	18 (69.2)	0.314

B, 3′-UTR			

Variable	Ins6/del6+del6/del6 n (%)	Ins6/ins6 n (%)	P-value

Age (years)			
≤70	74 (93.7)	5 (6.3)	
>70	9 (81.8)	2 (18.2)	0.064
Gender			
Male	42 (93.3)	3 (6.7)	
Female	41 (91.1)	4 (8.9)	0.987
Smoking status			
Non-smoker	51 (91.1)	5 (8.9)	
Smoker	32 (94.1)	2 (5.9)	0.601
Line of treatment			
1st-line	32 (94.1)	2 (5.9)	0.601
2nd-line or further	51 (91.1)	5 (8.9)	
Cycle			
≤4	59 (92.2)	5 (7.8)	
>4	24 (92.3)	2 (7.7)	0.985

**Table IV:** Association between polymorphisms and the efficacy of pemetrexed-based treatment in advanced NSCLC.

Genotype	Frequency n (%)	DCR n (%)	ORR n (%)
2R/2R+2R/3R	35 (38.9)	24 (68.6)	3 (8.6)
3R/3R+3R/4R	55 (61.1)	35 (63.6)	7 (12.7)
P-value	0.631	0.541	
Ins6/del6+del6/del6	83 (92.2)	55 (66.3)	10 (12)
Ins6/ins6	7 (7.8)	4 (57.1)	0 (0)
P-value	0.626	0.330	
2R/2R&6-/6+; 2R/3R&6-/6-; 2R/3R&6-/6+	28 (31.1)	20 (74.1)	3 (11.1)
2R/2R&6+/6+; 2R/3R&6+/6+	7 (7.8)	4 (57.1)	0 (0)
3R/4R&6+/6-; 3R/3R&6+/6-; 3R/3R&6-/6-	55 (61.1)	34 (62.5)	7 (12.5)
P-value		0.517	0.611

DCR, disease control rate; ORR, objective response rate.
